# Prevalence and impact of the *KIT* M541L variant in patients with mastocytosis

**DOI:** 10.18632/oncotarget.28614

**Published:** 2024-07-22

**Authors:** Luisa N. Dominguez Aldama, Eric Karlins, Xiaoping Sun, Daniel Veltri, Hirsh D. Komarow, Irina Maric, Dean D. Metcalfe, Melody C. Carter

**Affiliations:** ^1^Mast Cell Biology Section, Laboratory of Allergic Diseases, National Institute of Allergy and Infectious Diseases, National Institutes of Health, Bethesda, MD 20892, USA; ^2^Hematology Section, Department of Laboratory Medicine, Clinical Center, National Institutes of Health, Bethesda, MD 20892, USA; ^3^Bioinformatics and Computational Biosciences Branch, Office of Cyber Infrastructure and Computational Biology, National Institute of Allergy and Infectious Diseases, National Institutes of Health, Bethesda, MD 20892, USA; ^*^Co-first authorship

**Keywords:** mastocytosis, *KIT* M541L, *KIT* D816V, adults, pediatrics

## Abstract

Activating mutations in *KIT*, particularly D816V, have been associated with mastocytosis. Additionally, expression of heterozygous *KIT* M541L has been primarily reported in patients with pediatric mastocytosis. We thus examined the prevalence of this variant in pediatric and adult patients with mastocytosis (*n* = 100) compared to ancestry-matched 1000 genomes controls (*n* = 500) and patients with idiopathic anaphylaxis (*n* = 23). We then compared clinical symptoms and laboratory data on patients with systemic and cutaneous mastocytosis and bone marrow histopathology on a matched cohort with and without the *KIT* M541L variant. Overall, the *KIT* M541L variant was identified in 19 individuals; the majority were diagnosed with systemic mastocytosis (89.4%) with an associated *KIT* D816V mutation. There were no significant differences in peripheral blood parameters between groups. Patients with mastocytosis carrying the *KIT* M541L variant did not demonstrate significant differences in symptomatology compared to a matched reference cohort (*n* = 13/81) without *KIT* M541L. In patients with idiopathic anaphylaxis, no significant associations were observed. This study uniquely examines the prevalence and impact of the *KIT* M541L variant in both adult and pediatric patients with mastocytosis further stratified by disease variant. To our knowledge, this is the first case/control study to show a significant genetic association with mastocytosis at the *KIT* M541L locus.

## INTRODUCTION

Mastocytosis evolves from the clonal proliferation and occurrence of pathogenic mast cells (MCs) in skin and non-cutaneous organs and has largely been studied in individuals of European ancestry [1]. This disease is variously expressed in the skin as maculopapular cutaneous mastocytosis (MPCM), mastocytoma (MTOMA), and diffuse cutaneous mastocytosis (DCM). It manifests systemically primarily as indolent systemic mastocytosis (ISM) and smoldering systemic mastocytosis (SSM). Although most patients with mastocytosis experience mast cell-mediator symptoms, these symptoms do vary depending on the extent of tissue involvement and mast cell (MC) burden. Characteristically, children have transient cutaneous mastocytosis (CM), while adults commonly suffer from chronic systemic mastocytosis (SM) along with typical cutaneous manifestations of disease.

KIT is a tyrosine kinase receptor expressed on the surface of MCs, melanocytes, germ cells, hematopoietic stem cells and gastrointestinal stromal cells. The dimerization of KIT by stem cell factor (SCF) impacts MC proliferation and differentiation. *KIT* D816V is the most common somatic variant associated with SM. *KIT* M541L is largely reported to be a germline variant, the result of a conservative substitution from methionine to leucine, which occurs in the transmembrane domain at codon 541 (c.1621A>C, p.M541L (exon 10)) [2]. *In vivo* findings suggest there may be an effect on proliferation and survival of MCs expressing *KIT* M541L [3]. Another study demonstrated that M541L did not result in phosphorylation associated with ligand- independent tyrosine phosphorylation of KIT as an indication of activation in children with CM. The ligand-independent phosphorylation was associated with other KIT mutations at exons 8, 9, 11 and 17 [4]. *KIT* M541L has been reported in unaffected parents of patients with CM, suggesting that alone this variant is likely insufficient to cause mastocytosis. However, one case report documented an adult patient with a germline heterozygous *KIT* M541L variant, skin lesions consistent with CM and bone marrow MCs with aberrant co-expression of CD2/CD25 that were negative for the *KIT* D816V variant without meeting criteria for SM [5]. Other studies found no difference in the genotype frequencies of this variant among cohorts of Caucasian patients with chronic myelogenous leukemia and aggressive fibromatosis when compared to healthy controls and thus, concluded improbable relevance to pathogenesis [2, 6]. Nonetheless, with a Combined Annotation Dependent Depletion (CADD) [7] score of 16, *KIT* M541L is predicted to be within the 10 % most deleterious substitutions in the genome [8].

In two genome-wide association studies (GWAS) of patients with mastocytosis compared with control populations, germline single nucleotide polymorphisms (SNPs) were identified in patients with CM and SM associated with non-KIT genes that have an influence on myeloid progenitor cell growth and transcription factors that may impact the risk of developing mastocytosis.[9, 10]. Neither study reported *KIT* M541L as significantly associated with the mastocytosis. GWAS typically employs an additive model. This might explain why *KIT* M541L has not been reported as significantly associated with CM or SM in published GWAS. However, as will be documented in our report, we found, *KIT* M541L to be significantly associated with mastocytosis. Specifically, ours is the first case/control study to show a significant association with the *KIT* M541L variant in both pediatric and adult patients with mastocytosis when patients are stratified by disease variant and genotype at the *KIT* M541L locus. We further found a higher prevalence of this variant in patients with systemic mastocytosis with an associated *KIT* D816V mutation.

## RESULTS

### Demographics

**Figure 1:**
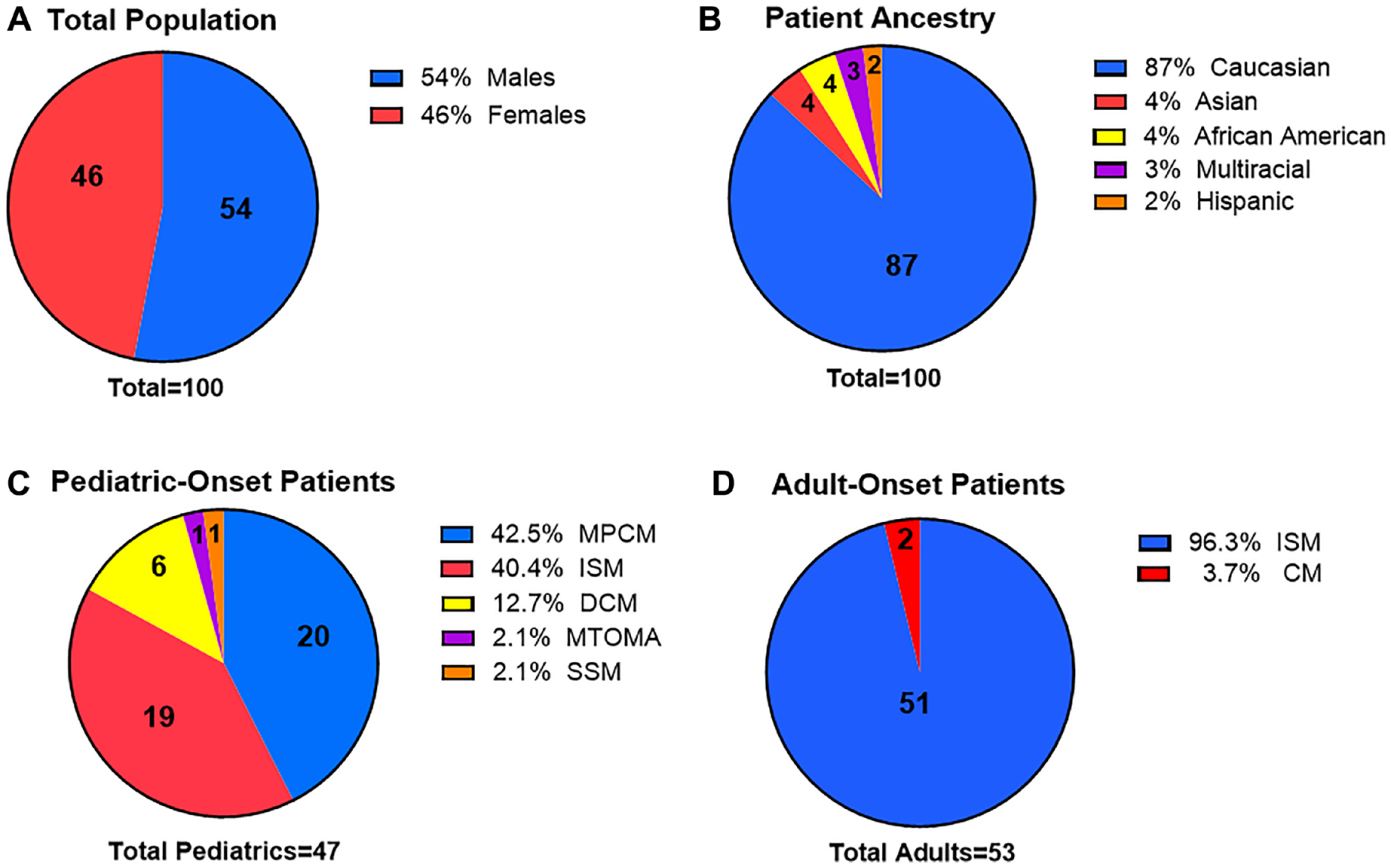
Mastocytosis patient demographics. (**A**) Total population by gender. (**B**) Total population by patient ancestry. (**C**) Diagnoses of pediatric-onset patients. (**D**) Diagnosis of adult-onset patients. Most of the patients have ISM (75%) and are Caucasian (87%). Abbreviations: SSM: smoldering systemic mastocytosis; DCM: diffuse cutaneous mastocytosis; ISM: indolent systemic mastocytosis; MPCM: maculopapular cutaneous mastocytosis; MTOMA: mastocytoma; CM: cutaneous mastocytosis.

The total mastocytosis cohort consisted of 54 males and 46 females (Figure 1A). By self-report, most patients were Caucasian (87%), followed by Asian (4%), African American (4%), Multiracial (3%), and Hispanic (2%) (Figure 1B). In the cohort of pediatric-onset patients *n* = 47 (47%), 20 had MPCM (42.5%), 19 had ISM (40.4%), 6 had DCM (12.7%), 1 had MTOMA (2.1%), and 1 had SSM (2.1%) (Figure 1C). Approximately, ninety-six percent of the adult-onset patients were diagnosed with ISM (Figure 1D). Overall, most patients had SM (70%) (Figure 1C, 1D). This cohort was compared to 500 matched controls without mastocytosis from the 1000 Genomes Project database.

### Ancestry data

Principal component analysis (PCA) was used to determine ancestry and match population-based controls to our mastocytosis cases. As seen in the Supplementary Figure 1, most patients with mastocytosis (in red) are classified as European. The remaining patients are represented in decreasing counts as American, South Asian, African, and East Asian ancestry. The results show that our patient cohort correlated ancestrally with the European group, which is reflective of our NIH population group with mastocytosis and the *KIT* M541L variant.

### Variant associations

The cohort was divided into heterozygous *KIT* M541L (15 patients, AC), homozygous alternate *KIT* M541L (4 patients, CC) and homozygous reference without *KIT* M541L (81 patients, AA), and subdivided by gender, disease variant, disease onset and presence of *KIT* D816V in peripheral blood (PB). The heterozygous group (AC) included 8 (53%) females and 7 (47%) males, the homozygous alternate (CC) had 1 (25%) female and 3 (75%) males, and the homozygous reference (AA) had 37 (46%) females and 44 (54%) males, (Table I). The predominant mastocytosis variant in the heterozygous genotype was ISM (86.6%) while the remainder was MPCM (13.4%). Additionally, the heterozygous cohort was represented by adult and pediatric patients with mastocytosis with 66.6% and 33.4%, respectively. As shown in Table I, disease variant for the homozygous alternate genotype was found only in patients with systemic disease and predominately in adults (75%). In contrast, the homozygous reference genotype group consisted of 65% ISM, 25% MPCM, 7.4% DCM, 1.3% SSM, and 1.3% MTOMA (Table I). There was a higher prevalence of ISM in all groups compared to cutaneous disease, which was not associated with age of onset (pediatric vs. adult).

**Table I:** *KIT* M541L genotypic summary

M541L genotype	Gender *n* (%)	Disease variant *n* (%)	Disease onset *n* (%)	PB KIT D816V *n* (%)
Heterozygous (AC) TOTAL: 15	8 (53) F 7 (47) M	13 (87) ISM 2 (13) MPCM	10 (67) A 5 (33) P	13 (87) Yes 2 (13) No
Homozygous Alternate (CC) TOTAL: 4	1 (25) F 3 (75) M	4 (100) ISM	3 (75) A 1 (25) P	4 (100) Yes
Homozygous Reference (AA) TOTAL: 81	37 (46) F 44 (54) M	53 (65) ISM 20 (25) MPCM 6 (7.4) DCM 1 (1.3) SSM 1 (1.3) MTOMA	40 (49) A 41 (51) P	43 (53) Yes 38 (47) No^*^

Abbreviations: A: Adult; DCM: diffuse cutaneous mastocytosis; F: female; ISM: indolent systemic mastocytosis; MPCM: maculopapular cutaneous mastocytosis; M: male; MTOMA-mastocytoma; P: pediatric; SSM: smoldering systemic mastocytosis. AC and CC cohorts have the M541L variant in at least one allele, AA cohort does not have the variant. ^*^Two patients with *KIT* D816Y, one with *KIT* F522C, and the remainder have no additional KIT mutations.

**Figure 2:**
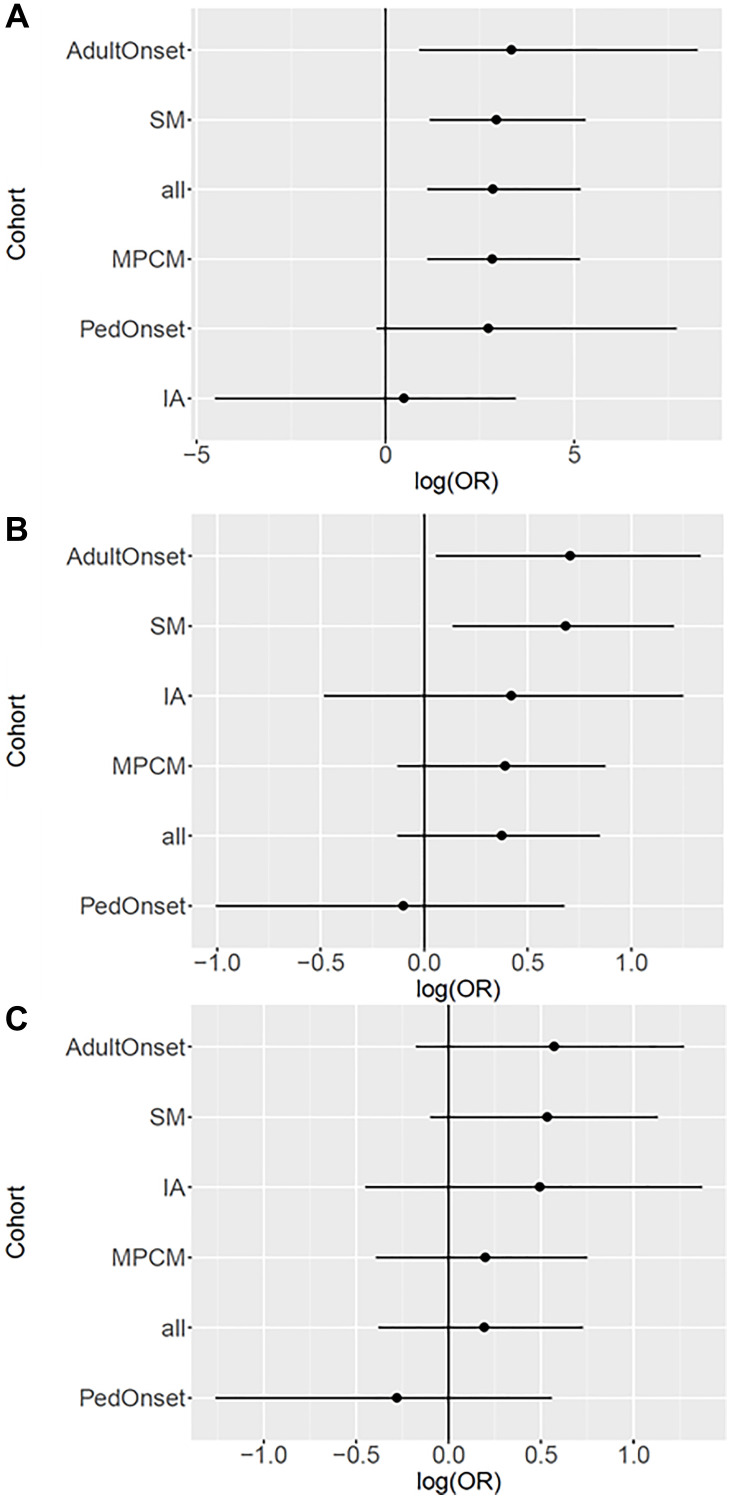
Log of odds ratios for genetic models in mastocytosis cohorts stratified by phenotype. For all models, the cohorts are sorted in descending order of the OR. The vertical line at log(OR) = 0.0 indicates no association with the disease. The horizontal line is the confidence interval. (**A**) Recessive model-The plot illustrates the log of the odds ratios (OR) for the recessive model in cohort studies. The OR represents the relative odds of disease for individuals carrying two minor alleles compared to those with zero or one minor allele. (**B**) Additive model-The plot displays the log of the odds ratios (OR) for the additive model in cohort studies. The OR represents the relative odds of disease for each additional minor allele. (**C**) Dominant model-The plot shows the log of the odds ratios (OR) for the dominant model in cohort studies. The OR represents the relative odds of disease for individuals carrying the minor allele compared to those with the major allele.

In our stratified analyses, each run on three inheritance models (additive, dominant, and recessive), we observed 5 significant associations after adjusting for multiple testing (adjusted *p* < 0.05) between *KIT* M541L. For the all mastocytosis group, the recessive model (Figure 2A) revealed a significant association with an odds ratio (OR) of 17.1 (95% confidence interval (CI): 3.1–175.5, FDR adjusted *p*-value: 0.0073). Similarly, in the MPCM group that included both adults and pediatric-onset patients, the recessive model showed a significant association with an OR of 16.9 (95% CI: 3.0–172.4, FDR adjusted *p*-value: 0.0073) (Figure 2A).

Furthermore, in the systemic group, the recessive model (Figure 2A) yielded a significant association with an OR of 18.8 (95% CI: 3.3–198.9, FDR adjusted *p*-value: 0.0073), as did the additive model (Figure 2B) with an OR of 2.0 (95% CI: 1.1–3.3, FDR adjusted *p*-value: 0.05). In the adult-onset group, the recessive model (Figure 2A) yielded a significant association with an OR of 28.2 (95% CI: 2.5–3874.4, FDR adjusted *p*-value: 0.0250). In the pediatric onset and idiopathic anaphylaxis groups, no significant associations were observed in any of the three genetic models (Figure 2A–2C). There is a higher prevalence of ISM in the cohort of patients with *KIT* M541L as demonstrated in both the recessive and additive models (Figure 2A, 2B) (Table I).

Across all samples the relative risk of mastocytosis for the CC genotype is 4.93 (95% CI: 3.05–7.98), while relative risk for the AC genotype is 0.96 (95% CI: 0.58–1.60). In the systemic disease cohort, risk of mastocytosis for the CC genotype is 5.21 (95% CI: 3.16–8.61), while relative risk for the AC genotype is 1.23 (95% CI: 0.71–2.12). We show significant associations in the recessive model for several of our stratified analysis. Overall, these results indicate that *KIT* M541L genotypes are associated with mastocytosis, as well as specific phenotypic groups within the mastocytosis cohort, namely, systemic disease with MPCM and adult onset. These findings suggest a potential role of this genetic variant in the etiology of mastocytosis as well as a contribution to the risk of a diagnosis of mastocytosis.

Six additional pediatric patients (1 CC, 5 AC) were identified with the *KIT* M541L variant by genotyping the pediatric cohort using NGS for KIT and non-KIT mutations (Supplementary Table 1). Four of six had SM, three with an additional *KIT* D816V variant and one homozygous (CC) for the *KIT* M541L with no other KIT variants. This data further supports a higher prevalence of the *KIT* M541L variant in SM.

### Clinical symptoms

Mast cell mediator symptoms, bone marrow histopathology and organomegaly were examined. Symptoms included flushing, pruritus, hives, abdominal pain, GERD, diarrhea, vomiting, joint and bone pain, fractures, osteoporosis, and headaches. Patients with the *KIT* M541L variant had proportionately more cutaneous, GI, neurologic and musculoskeletal symptoms when compared to the homozygous reference group without the mutation. In addition, the homozygous group with the *KIT* M541L on both alleles had less splenomegaly compared to the other groups, however, none of these differences reached significance. (Supplementary Figure 2).

### Laboratory and bone marrow (BM) findings

Seventy of 100 patients were diagnosed with SM. The PB ASqPCR was positive in 60/70 of these cases with SM (Table I). The PB ASqPCR for the *KIT* D816V mutation was negative for patients with CM (*n* = 29), and 9 patients in the SM cohort (2 D816Y, 1 F522C, 6 SM +BM). In patients with SM and the *KIT* M541L variant (both heterozygous (AC) and homozygous (CC)), all had the *KIT* D816V variant. The six patients with ISM and a PB KIT of <0.03 had the following characteristics; low bone marrow MC burden and/or without meeting the major criterion of >15 MCs per aggregate in their bone marrow biopsy and a sBT <20 ng/ml. The median age of disease onset was 25 years (range 0–64 years) and 36 years (range 0–60 years) in the heterozygous (AC) and homozygous alternate (CC) groups, respectively. The *KIT* M541L variant was mostly documented in patients with SM and the *KIT* D816V mutation.

Patients with a heterozygous *KIT* M541L, had a median serum tryptase of 31.7 ng/mL (range 1.8–222 ng/mL) (Table IIA). In comparison, patients with homozygous alternate for *KIT* M541L had median serum tryptase value of 33.4 ng/mL (range 23.4–40 ng/mL) and the homozygous reference group had a median value of 18.6 ng/ml (range 2–590). All median laboratory values in Table IIA are based on the normal level considering age and gender except for sBT (<11.5 ng/mL) and the positive PB *KIT* D816V (>0.03). The median alkaline phosphatase was 96 U/L for the heterozygous group (range 63–261 U/L), 95 U/L for homozygous alternate (range 52–259 U/L) and 82 in the homozygous reference group (Table IIA). There was no significant difference in median values for IgE, or B12. Thus, there were no significant differences in PB values between the heterozygous, homozygous, or homozygous reference groups for reference lab values.

**Table IIA:** Median peripheral blood values

M541L genotype	sBT ng/mL (range)	Alkaline phosphatase U/L (range)	IgE IU/mL (range)	B12 pg/mL (range)	PB KIT D816V (range)
Heterozygous (AC) TOTAL: 15	31.7 (1.8–222)	96 (63–261)	6.5 (1.9–238)	563^[*]^ (276–1493)	0.868^[†]^ (0.05–18.98)
Homozygous Alternate (CC) TOTAL: 4	33.4 (23.4–40)	95 (52–259)	10.5 (4.6–31.5)	534 (216–847)	2.621 (0.18–19.96)
Homozygous Reference (AA) TOTAL: 81	18.6 (2–590)	82^[‡]^ (46–474)	13.3 (0.9–1755)	572^[§]^ (206–4931)	0.744^[¶]^ (0.035–45.55)

^[*]^Not done-1/15; ^[†]^Negative-2/15, (Positive but unable to quantify-2/15); ^[‡]^Not done-1/81; ^[§]^Not done-7/81. ^[¶]^Negative-38/81 (Positive but unable to quantify- 6/81). AC and CC have the *KIT* M541L mutation in at least one allele whereas AA alleles do not have the mutation. Normal range for sBT (≤11.5) and the positive PB *KIT* D816V (>0.03) in all groups.

A median value of 0.868 for *KIT* D816V in PB was calculated for 11/15 heterozygous patients. The remaining patients were either negative or had positive results that were unable to be quantified (*n* = 4). All cohorts in the homozygous group were positive for *KIT* D816V with a median value of 2.621 (Table IIA). Therefore, the presence of a *KIT* M541L did not affect the PB D816V allelic burden in patients with systemic disease.

**Figure 3:**
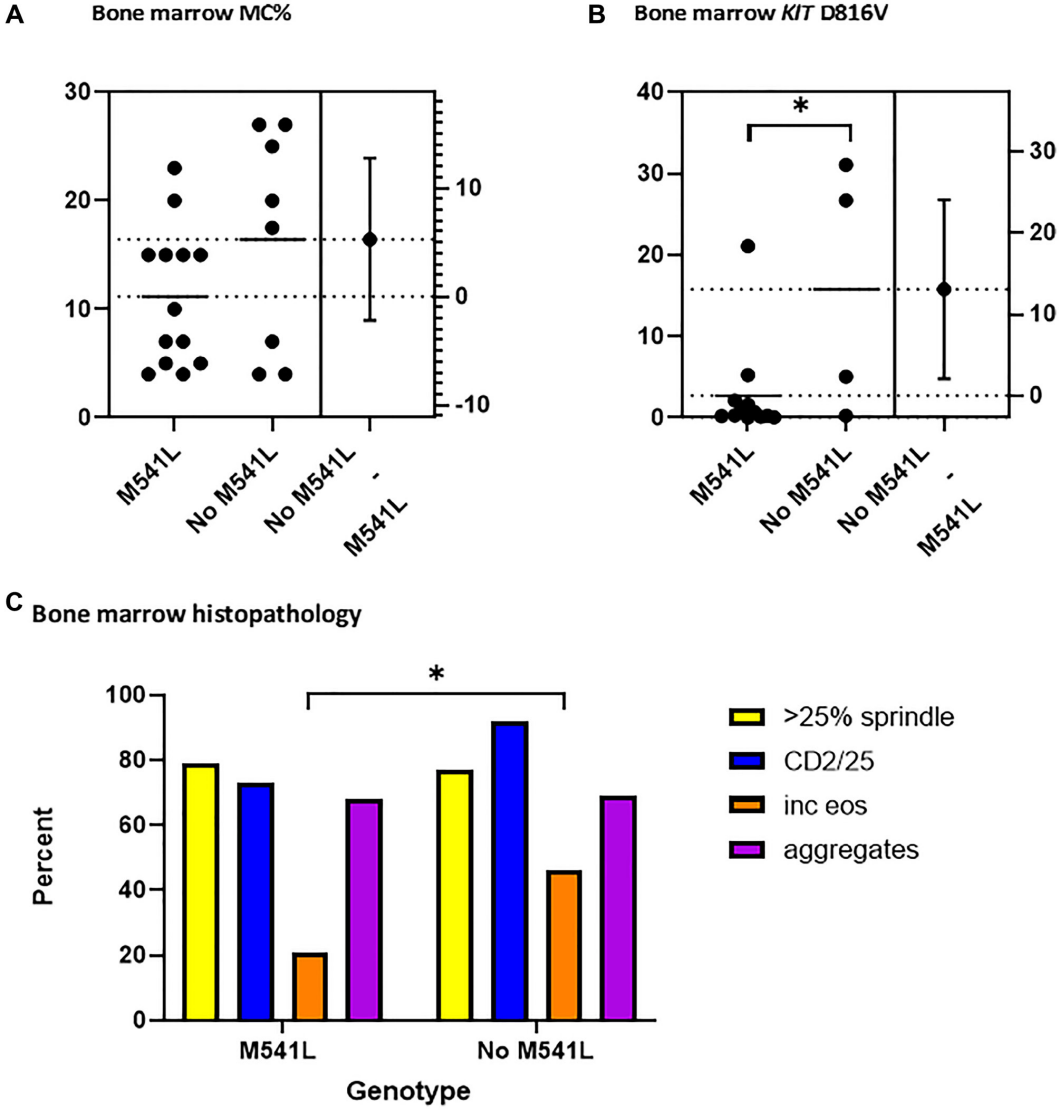
Bone marrow histopathology and KIT M541L variant. (**A**) No significant difference was demonstrated when bone marrow mast cell percent was compared in patients with (*n* = 13) and without (*n* = 8) the KIT M541L variant. (**B**) Patients with the KIT M541L variant (*n* = 14) had a significantly lower allelic burden of the KIT D816V mutation when compared to patients without the KIT M541L (*n* = 4). (**C**) Patients with the KIT M541L variant had significantly lower eosinophils when compared to patients without the KIT M541L variant. No difference in the clonal mast cell population parameters ((CD2/25+MCs, >25% spindle-shaped MCs, MC aggregates, or >15 MCs).

Bone marrow histopathology was compared between patients heterozygous or homozygous for *KIT* M541L (AC *n* = 15, CC *n* = 4) and the homozygous reference patients (AA *n* = 13). There were approximately equal numbers of pediatric and adult patients in the homozygous reference group to reflect the distribution of the total group (Table I). The biopsies were analyzed for the presence of MC aggregates, MC infiltrate in total cellularity, spindle-shaped MCs, and CD25+/CD2+ expression by MCs. Median percentage of MCs was lower for the homozygous group at 5.5%, but this only accounts for 3 of 4 patients (Table IIB). Table IIB also shows additional non-KIT mutations found in patients. One patient with heterozygous M541L variant also carried JAK2, CALR and MPL variants. Although there was no significant difference in MC burden (Figure 3A), there was a significantly higher allelic burden in the bone marrow from patients without the additional *KIT* M541L mutation (p 0.01, Figure 3B). Additionally, patients in this same cohort had a significant increase in BM eosinophils (*p* 0.02, Figure 3C). The higher allelic burden and eosinophil presence may reflect an increase population of a mutant MCs in patients without a concurrent M541L variant.

**Table IIB:** Median bone marrow histopathology

M541L genotype	MC aggregates	Median % MCs (range)	>25% Spindle- Shaped MCs	CD2+/ CD25+	↑ Eos	Perivascular aggregates	Paratrabecular aggregates	Median BM KIT D816V (range)	Non-KIT Mutations
Heterozygous (AC) TOTAL: 15	11/15	15% (4–22.5)	12/15	11/15	1/15	5/15	4/15	0.592, 9/15 (0.01–21.07)	JAK2, CALR and MPL, 1/15
Homozygous Alternate (CC) TOTAL: 4	3/4	5.5% (5–10)	4/4	4/4	1/4	2/4	1/4	1.15, 2/4 (0.70–1.60)	None
Homozygous Reference (AA) TOTAL 13	9/13	18.7% (4–30)	10/13	12/13	6/13	11/13	9/13	15.9, 4/13 (0.25–31.08)	None

Abbreviations: BM: bone marrow; MC: mast cell. AC and CC have the *KIT* M541L variant in at least one allele whereas AA alleles do not have the *KIT* M541L variant. All non-KIT mutations (JAK2, CALR and MPL) represent one subject in the heterozygous group with concomitant primary myelofibrosis.

In the cohort of pediatric-onset patients analyzed by NGS (*n* = 69), 6 additional patients were identified with the *KIT* M541L variant (Supplementary Table 1). One patient with SM and the homozygous variant of *KIT* M541L did not have an additional KIT mutation. Thus, in agreement with the findings in this principal cohort, the majority had SM with the *KIT* D816V variant.

## DISCUSSION

We found a significant association between *KIT* M541L genotype and the diagnosis of mastocytosis. Individuals carrying the CC homozygous genotype at the *KIT* M541L locus are predicted to have an almost 5-fold increased risk of mastocytosis onset compared to individuals with any other *KIT* 541 genotype. Though our cohort size is small and is even more diluted when we stratify into subpopulations by phenotype, our results indicate that this *KIT* M541L association is largely driven by patients with systemic disease. *KIT* M541L genotype seems to be important in risk of SM, though further studies will need to be performed to confirm these results. The *KIT* M541L genotype also shows a significant association with adult-onset disease. This is not unexpected, since 96% of our adult-onset cases had systemic disease. Pediatric onset mastocytosis patients are typically diagnosed with CM, whereas adults are diagnosed with SM with cutaneous manifestations [11]. The *KIT* M541L genotype was not found to have a significant association with risk in the IA or pediatric onset phenotypic groups; however, it may have a role in pediatric onset disease with SM.

We identified heterozygous and homozygous recessive *KIT* M541L variants in 19 adult and pediatric patients with cutaneous and systemic mastocytosis. Using ancestry matched controls to our case cohort at a 5 to1 ratio, we found a significant association (FDR adjusted *p* = 0.0073) in the recessive model. The overall allele frequency of the KIT M541L variant in our patient cohort was 11.5%, which is slightly higher than in the general population [2]. However, most patients (89.4%) with *KIT* M541L variant were diagnosed with ISM and had concurrent *KIT* D816V mutation. Interestingly, one patient with ISM and a homozygous *KIT* M541L lacked additional *KIT* mutations.

A 2021 GWAS by Galata et al. reports several loci significantly associated with SM. [9] *KIT* M541L is not one of the loci reported. Looking through their summary statistics, the association at *KIT* M541L in their study would be significant by our threshold but does not reach genome-wide significance (*p* = 0.01). This is comparable to what we see in the additive model in the systemic group in our study. Galata et al. do not report findings from the recessive model. It is possible that if they applied the recessive model *KIT* M541L would be amongst the loci that they report as significant.

*KIT* M541L is a polymorphism found in 5–9% of the general population in subjects with varied ancestral backgrounds with the highest percentages in Southeast Asia (9%) and Europe (8%), according to the 1000 Genomes Project [12]. We see a difference in the allele frequency between our cases and our ancestry-matched controls. The allele frequency in all mastocytosis cases is 11.5% and in systemic mastocytosis 14.9%, while in the ancestry-matched controls, the allele frequency is 8.3%. Our patients with mastocytosis mapped to the European control population with a higher frequency compared to other ancestral data points. Indeed, our patient cohort at the NIH mirrors this distribution with 92% having European ancestry [1] and supports our exploration in this population.

Foster et al. first noted the heterozygous expression of this variant in subjects from two families, pairs of identical twins with CM and one parent, concluding it may predispose to pediatric mastocytosis [3]. Subsequently, other studies recorded the presence of this variant from skin biopsies in children with mastocytosis. Bodemer et al. reported 28% of patients with CM had *KIT* M541L and none with the wild-type codon 816 sequence carried the *KIT* M541L variant [4]. A supplemental study of the same cohort, with an additional 16 patients, found no new cases with this variant [13]. Additionally, Rocha et al. reported M541L in a skin biopsy in an adult female with abnormal BM mast cells [5]. Our study demonstrated a parallel finding in an inverse pattern as in the Bodemer et al. study with *KIT* M541L variant demonstrating an association with *KIT* D816V variant. However, the patients in the Bodemer et al. study did not report a diagnosis of SM. In agreement with the Foster et al. study, the variant is a germline genotype.

We explored clinical manifestations to determine if there was a disease-modifying effect of *KIT* M541L. Although the peripheral blood indices and overall bone marrow histopathology did not demonstrate a significant difference, there was a higher allelic burden for *KIT* D816V in the BM MCs in patients without the *KIT* M541L variant. Further, there also may be a disease-modifying effect on the bone marrow pathology demonstrated by a higher eosinophil presence, which needs to be verified in a larger sample. If indeed there is a higher mutant MC population that does not correlate with clinical symptom manifestations, MC cytoreductive therapy may not be as effective for a symptomatic response in a group with the *KIT* M541L variant. We found that splenomegaly was not seen in patients in the homozygous alternate group while 32% and 15% of patients in the heterozygous and homozygous reference groups, respectively, had splenomegaly. Thus, patients with a *KIT* M541L homozygous alternate mutation appear to have less extramedullary tissue burden in the presence of a higher allelic burden.

Our findings demonstrate *KIT* M541L at a higher frequency in patients with SM. Systemic disease typically involves a *KIT* D816V somatic mutation and is often missed since WES/WGS screening for rare variants do not include the parameters to capture this variant. The *KIT* M541L variant was to some extent more prevalent in our patient cohort compared to the general population. In patients with mastocytosis, in some instances, *KIT* M541L may be disease-modifying. demonstrated by a higher allelic burden of *KIT* D816V mutation patients without the *KIT* M541L variant. In addition, patients homozygous for the *KIT* M541L variant did not have a history of splenomegaly and less splenomegaly was seen in the heterozygous group, although not significant. Therefore, screening for this mutation in patients with mastocytosis may have some value for targeted therapy, (symptomatic vs. cytoreductive), however, larger numbers are needed for proof of concept. This investigation is the largest study to date of *KIT* M541L variant in both adults and pediatric patients with cutaneous and systemic disease, and the first to document a homozygous mutation in a patient that met criteria for systemic disease without an additional KIT mutation.

## MATERIALS AND METHODS

### Study participants

Participants were enrolled in a NIH IRB-approved protocol (NCT00044122) after informed consent for parents and assent for children >6 years of age. This protocol allows for a longitudinal study focused on MC survival and proliferation and disease manifestations. Mastocytosis age of onset ranged from birth to approximately 72 years diagnosed using the WHO criteria for CM and SM [14]. Of the 100 patients analyzed for this study (81 case controls, 19 with the *KIT* M541L variant). Forty-seven patients with pediatric-onset disease from birth to age 18, were diagnosed with either ISM, MPCM, MTOMA, DCM or SSM. Fifty-two adult-onset patients, ages 18 to 72, were diagnosed with ISM and one adult with CM. Patients (age 13–69 years) with idiopathic anaphylaxis (*n* = 23) (IA) defined by current guidelines [15] were enrolled on an IRB-approved protocol (NCT00719719). The median age of these patients was 43 years. Sixty six percent were female and 93% of patients were Caucasian. Patients in both groups underwent a clinical history, physical examination, and routine laboratories to screen for liver enzymes, serum baseline tryptase (sBT), alkaline phosphate, B12, immunoglobulins, and complete blood counts and differential. Additionally, patients with mastocytosis were assessed for organomegaly and MC-mediator symptoms. When indicated, an allele-specific qPCR for the detection of *KIT* D816V mutation in peripheral blood (PB) was utilized, along with a bone marrow biopsy to confirm the diagnosis of ISM based on the WHO classification of myeloproliferative neoplasms [14]. Patients self-identified their racial/ethnic group and were ancestrally and genetically matched to controls from European, Southeast Asian, East Asian, African, and Admixed American (North, Central and South America) ancestries using the 1000 Genomes Project database [12].

### Total serum tryptase laboratory data measurements

Total sBT was determined using a fluoroenzyme immunoassay (Phadia Immuno CAP, Uppsala, Sweden) at a CLIA-approved lab (Mayo Medical Labs, Rochester, MN, USA). sBT values and clinical labs for each case were performed through the Clinical Center CLIA-approved lab, then compiled in the Clinical Research Information System (CRIS), an electronic medical record for NIH patients. Laboratory data were selected from CRIS to match the blood collection date for exome sequencing. The normal reference range for the tryptase assay is 0.00–11.5 ng/ml.

### *KIT* D816V mutation allele-specific qPCR assay

The *KIT* D816V allelic burden was quantified from genomic DNA from peripheral blood (PB) and/or bone marrow (BM) using allele-specific qPCR (ASqPCR), as described [16]. The testing was conducted in duplicate, and the assay was considered positive with values of *KIT* D816V mutation positive cells ≥0.01%.

### Bone marrow preparation and flow cytometry

In-house bone marrow biopsies were fixed, paraffin-embedded and processed for morphological evaluation as described [17]. Immunohistochemical staining using anti-tryptase, anti-CD117 (Cell Marque, Hot Springs, AR) and anti-CD25 antibodies (Vision BioSystems, Norwell, MA, USA) was performed using an automated stainer (Ventana Ultra). Multi-parameter flow cytometry for mast cell analysis was performed on aspirates using anti-CD2, anti-CD25, anti-CD45 and anti-CD117 antibodies [17]. Data was analyzed using FCS Express (DeNovo Software, Pasadena, CA, USA).

### Sequencing and CMA methods

#### Whole exome sequencing (WES)

Informed consent for WES was obtained from patients and/or parents on NIH-approved protocol (NCT 03206099). Research-based exome sequencing was performed on study participants (*n* = 100) as described in Supplementary Methods (details in the Online Repository) [18]. Relevant findings were confirmed by Sanger sequencing or other appropriate methods meeting Clinical Laboratory Improvement Amendments/College of American Pathologists (CLIA/CAP) requirements. Genotypes for our patients were extracted from the VCF file at the *KIT* M541L locus (GRCh37 4:55593464-A-C). The patients heterozygous for *KIT* M541L are represented by AC, homozygous for *KIT* M541L or homozygous alternate represented by CC, and patients without a *KIT* M541L mutation homozygous reference, AA.

#### Next-generation sequencing (NGS)

Data from the QIAseq Targeted DNA panels were analyzed using the Biomedical Genomics Analysis module of the QIAGEN CLC Genomics Workbench software. An average quality score of 44 and UMI coverage of 2000X was used. The detected variants were interpreted using QIAGEN Clinical Insight interpret (QCI^®^) software by qBiomarker somatic mutation PCR array human KIT pathway and/or targeted NGS Human Neoplasms Panel.

### Statistical analysis

Unrelated probands were selected with exome sequencing data available (*n* = 100) to compare to matched controls from 1000 Genomes phase 3 (*n* = 2504). Exome genotype data from our patients were processed with GATK best practices (v4.2.0) [19] and merged with 1000 Genomes data. Principal component analysis (PCA) was performed using ancestry-informative markers [20]. To ensure robust comparisons, we stratified the samples based on different phenotypes, including all mastocytosis samples (*N* = 100), MPCM (*N* = 89), pediatric-onset (*N* = 47), adult-onset (*N* = 53), and systemic disease (*N* = 71). Cutaneous disease (CM) to include MCPM, DCM and MTOMA (*N* = 98) was not run as a separate cohort since it almost entirely overlaps with the “all mastocytosis” group. An idiopathic anaphylaxis cohort (*N* = 23) was also run as a comparison group. Each group of patients was separately ancestry-matched to 1000G controls in a 5 to 1 control: case ratio with the “pairmatch” function of the R package optmatch [21], using eigenvectors from principal components (PCs) 1 to 5. Genotypes for *KIT* M541L variant (GRCh37 4:55593464-A-C, rs3822214) from 1000 Genomes controls and our patients were combined and logistic regression association tests, using Firth’s bias reduction method (https://cran.r-project.org/web/packages/logistf/logistf.pdf), were performed using R (version 3.6.1), adjusting for PCs significantly associated with case/control status. For each stratified analysis, we ran three different models: additive, dominant, and recessive [22]. *P*-values were adjusted for multiple testing using the False Discovery Rate (FDR) method (18 tests in total) [23, 24] and considered significant if FDR adjusted *p* < 0.05. Statistical comparisons of clinical manifestations using the Fisher’s Exact Test and bone marrow histopathology by the Chi-square analysis, with a *p*-value of < 0.05 for significance, were performed using PRISM GraphPad (La Jolla, CA, USA).

## SUPPLEMENTARY MATERIALS



## Abbreviations

MC: mast cells
MPCM: maculopapular cutaneous mastocytosis
DCM: diffuse cutaneous mastocytosis
MTOMA: mastocytoma
ISM: indolent systemic mastocytosis
CM: cutaneous mastocytosis
SM: systemic disease
SSM: smoldering systemic mastocytosis
CADD: Combined Annotation Dependent Depletion
GWAS: genome-wide association studies
SNPs: single nucleotide polymorphisms
sBT: serum baseline tryptase
PB: peripheral blood
ASqPCR: allele-specific qPCR
WES: Whole exome sequencing
WGS: Whole genome sequencing
PCA: Principal component analysis
GERD: gastroesophageal reflux disease
NGS: next-generation sequencing
BM: bone marrow
CC: homozygous alternate
AC: heterozygous
CC: homozygous reference
