# Supplementary material for: Prevalence and impact of the KIT M541L variant in patients with mastocytosis
Source: Oncotarget. 2024 Jul 22;15:521–31. doi: 10.18632/oncotarget.28614 (PMC11262411; doi:10.18632/oncotarget.28614)
Supplement: Supplementary file 1 [file oncotarget-15-28614-s001.pdf]

Prevalence and impact of the *KIT* M541L variant in patients with mastocytosis

SUPPLEMENTARY MATERIALS

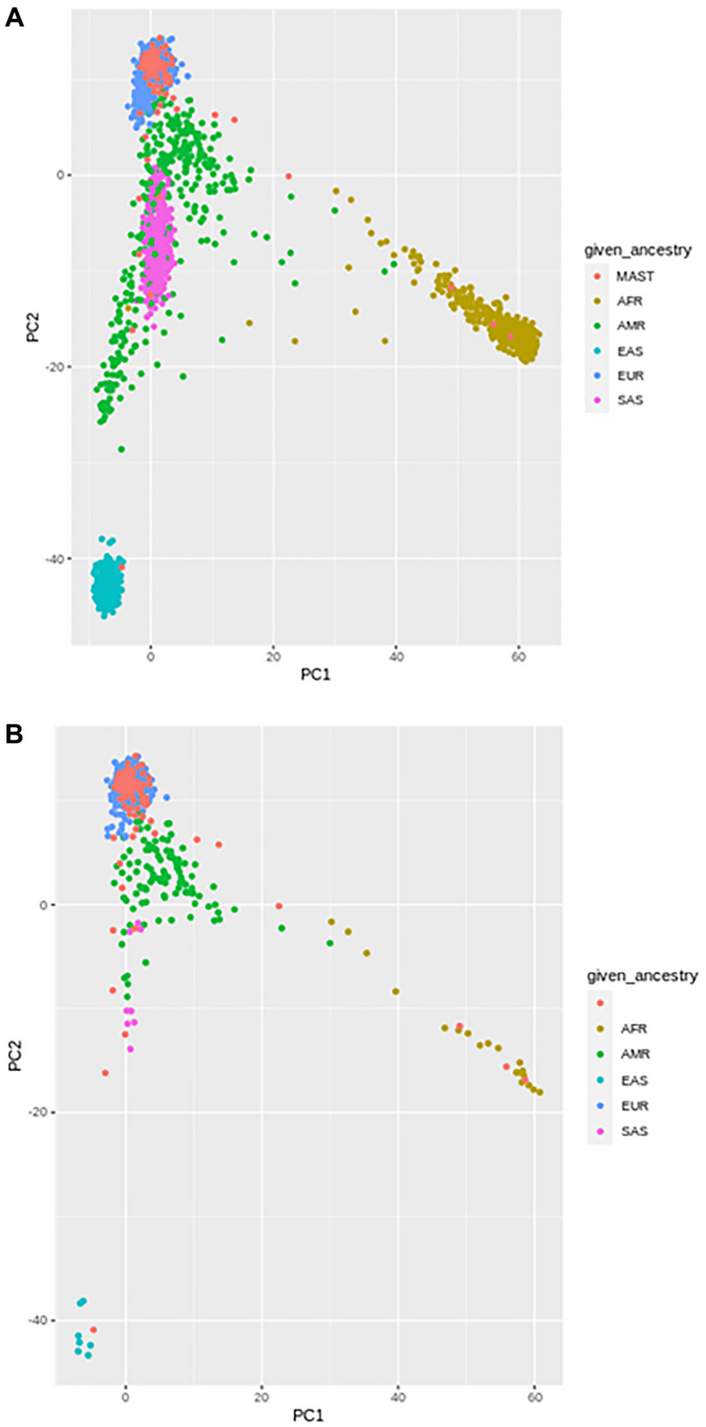

Supplementary Figure 1: PCA showing ancestry of cases and controls from the 1000 genomes project. The figure in part A shows before ancestry matching and part B shows after matching 5 controls to every 1 case. AFR-Africans, AMR-Americans, EAS - East Asians, EUR-Europeans, MAST- Mastocytosis, SAS-South Asians. The red dots correspond to our patients with mastocytosis while the remaining colors are for controls with varying ancestries. Most of the cohort with mastocytosis have European ancestry.

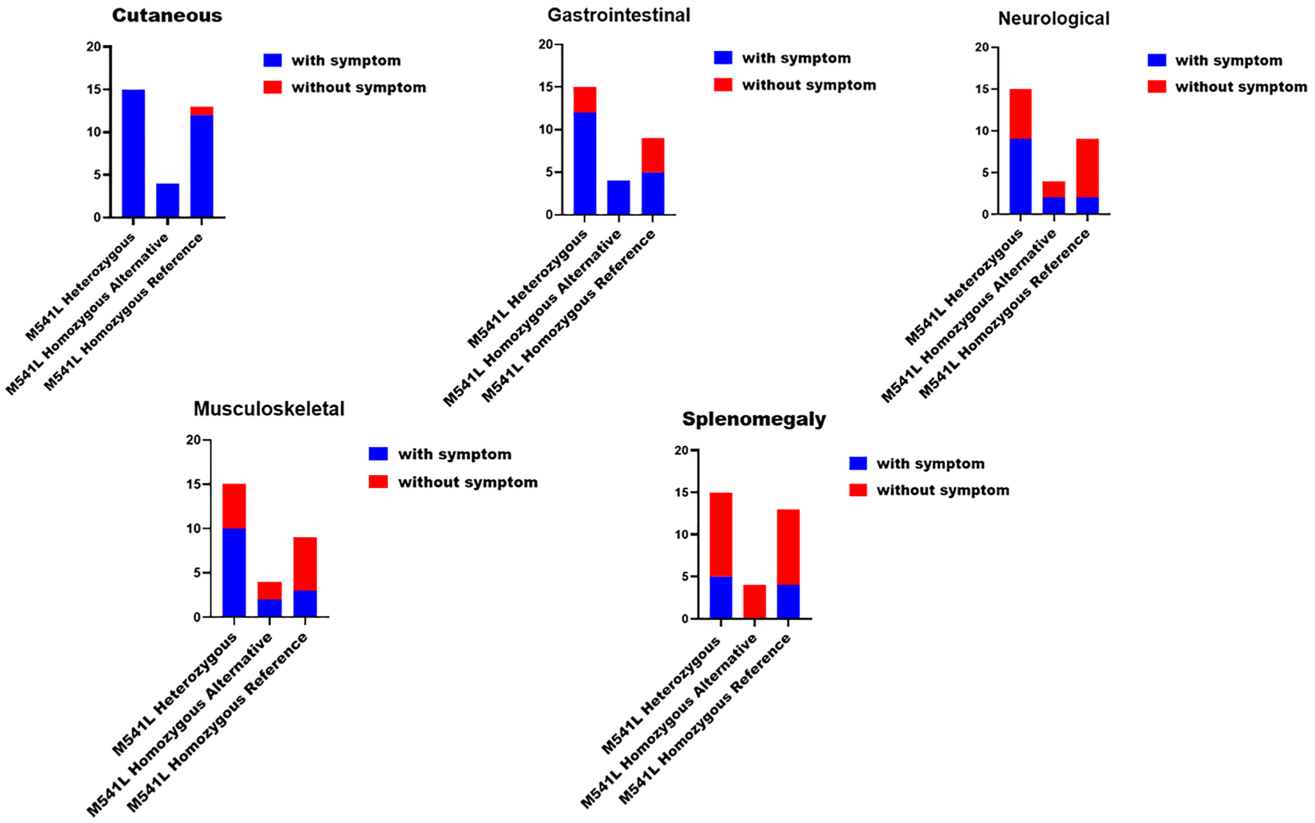

Supplementary Figure 2: Clinical manifestations of mastocytosis by genotype. There is a total of 19 heterozygous, 5 homozygous alternate and 13 homozygous reference cases. The heterozygous cohort symptoms reflect 100% cutaneous, 80% gastrointestinal, 53% neurological, 60% musculoskeletal, and 33% splenomegaly cases, the homozygous alternate reflect 100% cutaneous, 100% gastrointestinal, 60% neurological, 40% musculoskeletal, and no splenomegaly cases whereas the homozygous reference cohort has 70% cutaneous, 38% gastrointestinal, 15% neurologic, 23% musculoskeletal and 15% hepatosplenomegaly. There was no significance difference in clinical parameters but a trend for less symptoms in the two groups with the *KIT* M541L variant as well as less splenomegaly.

Supplementary Table 1: KIT M541L NGS genotype summary

M541L Genotype	Gender *n*	Disease variant *n*	Disease onset *n*	PB KIT D816V *n*
Heterozygous (AC)<br>TOTAL: 5	4 F<br>1 M	3 ISM<br>2 MPCM	5 P	3 Yes<br>2 No
Homozygous Alternate (CC)<br>TOTAL: 1	1 M	1 ISM	1 P	1 No

ISM-indolent systemic mastocytosis, MPCM-maculopapular cutaneous mastocytosis, AC and CC cohorts have the M541L variant in at least one allele.
